# Building research capital to facilitate research

**DOI:** 10.1186/1478-4505-11-12

**Published:** 2013-04-04

**Authors:** Gill Green, Melanie Rein

**Affiliations:** 1 School of Health & Human Sciences, University of Essex, Wivenhoe Park, Colchester CO4 3SQ, UK; 2 Self-employed Analytical Psychotherapist (Jungian), Cambridge, UK

## Abstract

The National Institute for Health Research, Research Design Service (NIHR RDS) was set up to increase the number and proportion of high quality applications for funding for applied and patient focused health and social care research. Access to specialist expertise and collaboration between researchers and health practitioners at the proposal development stage is crucial for high quality applied health research. In this essay we develop the concept of ‘research capital’ to describe the wide range of resources and expertise required to develop fundable research projects. It highlights the key role the RDS plays supporting researchers to broker relationships to access the requisite ‘research capital’.

## Introduction

**Figure 1:**
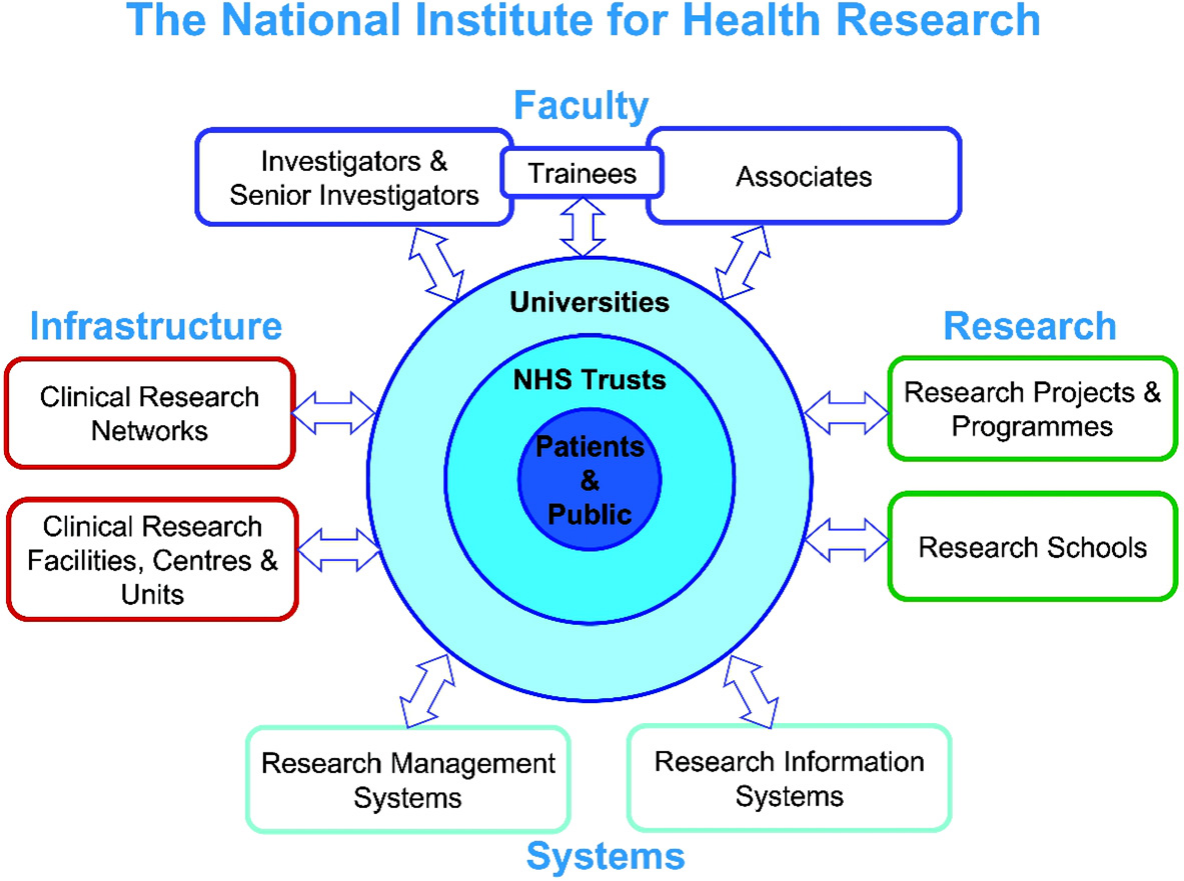
**The health research system of the NHS and the NIHR key work strands **[3].

National health research systems which harness the capabilities of key stakeholders and promote joined-up working between them, are likely to be the most effective [1]. The National Institute for Health Research (NIHR) was set up by the Department of Health in England to realise the unique research opportunities and potential within the NHS and to provide a framework to support the NHS as a national research facility. The central mission of the NIHR is “to create a health research system in which the NHS supports outstanding individuals, working in world class facilities, conducting leading edge research focused on the needs of patients and the public” [2]. To achieve this aim the NIHR established a range of research programmes and a research infrastructure of networks, centres, units and research systems to foster a supportive and robust environment within which research in the NHS can flourish (Figure 1).

Collaboration and partnership between researchers and decision-makers, and/or managers is seen as key for successful research and translation of research into practice [4-7]. In this essay we focus upon partnerships and support required to develop a robust research proposal. A number of clinicians and university researchers in England have developed strong applied health research programmes and access to extensive research networks. However, novice researchers who are based in an environment that lacks a rich research culture, may struggle to develop fundable research proposals. Part of the NIHR aim is to support health professionals who may have excellent ideas for research but lack the methodological expertise or access to the requisite partners to design and carry out high quality research. The NIHR Research Design Service (RDS) was set up specifically to address this and to offer support to less-experienced NHS researchers and those working in organisations without a research track record applying to the NIHR research programmes. There are 10 regional RDSs covering the whole of England and this article focuses upon the activity of the RDS for the East of England (RDS EoE). It develops the notion of ‘research capital’ and assesses the role of the RDS as brokers across ‘structural holes’ and as facilitators of ‘network closures’ in order that ‘research knowledge and capital’ is translated into high quality and effective research proposals.

### What is research capital?

By ‘research capital’ we refer to the resources and networks that are required to develop robust research.^a^ It draws on the theoretical concept of ‘social capital’, which was popularised by Robert Putnam [8,9] in his analysis of the decline of civic and community engagement and informal socialising in the United States. Whilst there are debates about what constitutes ‘social capital’, in most definitions the role of networks and civic norms are key and are often operationalized as social relations, formal and informal social networks, group membership, trust, reciprocity and civic engagement [10]. According to Portes [11], social capital is the access individuals have to scarce resources via their membership in networks or relationships with others. Social capital is usually understood to belong to the community or group rather than the individual and is generally associated with positive outcomes such as lower levels of income inequality and better health [12], higher educational achievement [13] and economic achievement [14]. Those with stronger ‘social capital’ are more likely to be “housed, healthy, hired and happy” [15]. While such networks provide certain opportunities for the group or community, ‘structural holes’ appear when knowledge held within groups is not shared across groups. Burt highlights this by saying: “information circulates more within than between groups, within work group more than between groups, within a division more than between divisions, within an industry more than between industries” [16]^b^.

Centres of research excellence within the health sector are generally characterised by a strong research track record, a critical mass of well-qualified and experienced researchers, excellent links with patients and the public, and access to research resources and infrastructure, *i.e.*, they have strong ‘research capital’. Those with stronger ‘research capital’ are more likely to develop research that is feasible, fundable and influential. However, not all researchers are based in centres of research excellence, nor do they have access to the requisite ‘research capital’ to facilitate the development of excellent research ideas into fundable projects.

### Building research capital

The overall aim of the RDS is to support: “researchers to develop and design high quality research proposals for submission to NIHR and other national, peer-reviewed funding competitions for applied health or social care research” [17].

The specific aims of the RDS are to: “Offer fit for purpose advice and support on research design and methodology to researchers making funding applications and doing research within the health research system; and to increase the number and proportion of high quality applications for funding for applied people and patient focused health and social care research” [17].

In practice, each RDS provides research advice on all aspects of developing a research proposal including feasibility of research; study design; qualitative and quantitative methodologies; statistics; health economics; clinical trials; and, patient and public involvement. Requests for research advice come through a number of different routes, some of which are dependent on relationships developed between RDS staff and individual researchers, others which result from the direct marketing activities of the RDS.

However, while the two aims above outline the work of the RDS, in reality its work is more complex and subtle. The RDS’s role is primarily to bridge the gap between practice and academia and the public; that is, to build ‘research capital’ by becoming brokers of knowledge across ‘structural holes’. This involves RDS staff working across three different groups: practitioner groups, who have experience and expertise in clinical practice and patient care; academic groups, usually based in universities who have expertise and knowledge in research methodologies undertaking research; and, patient groups who have the expertise and knowledge of patient experience.^c^ Thus, through its sophisticated network of partner organisations and its dynamic research capacity, the RDS provides a bridge which enables practitioners, academics, service users and other related individuals and organisations to come together to form new research partnerships.

Once a bridge has become established the RDS then supports the embryonic research team by facilitating a ‘closed network’ [16], forming a research team in which the knowledge and information remains generally within the boundaries of the team, in order that trust is developed, information controlled, and confidentiality maintained.

The NIHR RDS EoE itself is formed of a partnership which has been set up to bridge ‘structural holes’ across health and academic organisations in the East of England. Currently the NIHR RDS EoE has five university partners across the East of England and four NHS Trusts. The RDS EoE is also able to access other expertise and knowledge from within its wide network of contacts.

While specific health research expertise and knowledge is held in each of the five universities and NHS Trusts, it is the complex network of staff and organisational relationships which spans across and through the RDS EoE partnership which enables the building of bridges across ‘structural holes’.

For example, in a recent ophthalmic proposal for the NIHR Service and Delivery Organisation funding stream (now Health Services Delivery and Research), the RDS EoE engaged with the lead researcher, a hospital consultant, as he was submitting his outline application. The researcher had initially been awarded funding to support the writing of a proposal but made contact with the RDS EoE only two weeks before the submission deadline for his outline application. As time was limited, it was only possible to offer general advice on the outline bid although once the outline application was accepted, the RDS EoE then went on to play a major role in supporting the research proposal. The Research Adviser helped to redesign the proposal by advising on specific methodological areas and also by facilitating contact with specialist researchers, *e.g.*, a health economist and a statistician. The Research Adviser also arranged discussions with a newly set up public and patient group and facilitated contact with a clinical trials unit. In collaboration with the local trust R&D office, the Research Adviser worked with the researcher in order that his original costing would accurately represent the work involved in the research.

**Figure 2:**
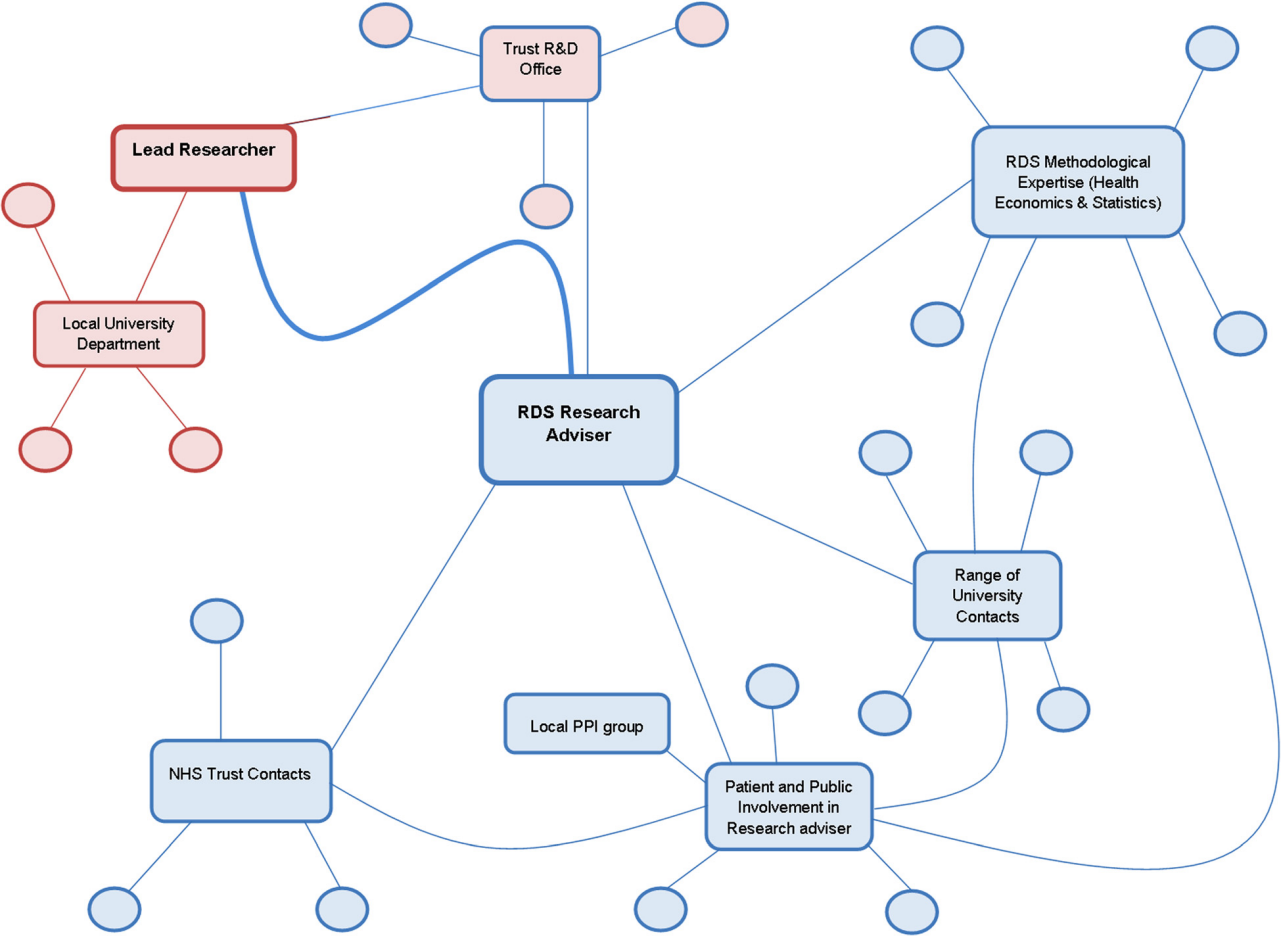
**Network linking researcher to wider networks via the RDS (adapted from Burt) **[18].

As this example shows, the RDS EoE builds ‘research capital’ to support the development of the research proposal by (1) providing methodological expertise; (2) supporting and developing research partnerships between clinicians and academics; and, (3) enabling and supporting the inclusion of patients and the public in the research process in order that they become active partners in the research. Figure 2 illustrates the connections which the researcher has to various organisations in contrast to the connections of the Research Adviser.

While (1) above may often include expertise within the RDS EoE, it may also require access to more specialised research knowledge. Access to this type of specialised research knowledge is not necessarily easily available to the research practitioner, or even to researchers within specific research organisations. However, with the increasing network and connections made by individuals within the RDS EoE with academic and research groups, access to these specific groups becomes more accessible to the researcher. Coupled with this emerging network is the more specific knowledge which the RDS EoE holds on research taking place throughout the region; as the number of research projects which the RDS EoE supports grows, so the knowledge of who is doing what research within the region becomes more accessible enabling the RDS EoE to build bridges and connections across the various research groups and research individuals within the region. It thus plays a vital part in building ‘research capital’ in the region by providing access to expertise and ‘bridging structural holes’ in order to build a system within which robust research proposals can be developed.

Many clinicians and applied health researchers in the EoE have successful research careers, which were established long before the RDS existed and have access to sufficient ‘research capital’. However, clinicians without a research track record may need support, and occasionally more experienced researchers may also need support to access specific methodological expertise or to contact new partners, such as to obtain feedback on the proposed research from a particular patient group, or an experienced university-based researcher may be seeking a clinical partner. The RDS can thus provide assistance to a range of applied health researchers.

The national network of RDSs thus plays a pivotal role in providing researchers access to the requisite ‘research capital’ by bridging ‘structural holes’ and developing networks. It harnesses the knowledge of the clinician with the research skills of the academic and the lived experience of the patient. This ensures that the health research skills and experience held within the knowledge economy are provided with the supportive infrastructure to develop fundable applied health research of relevance to the patient.

## End notes

^a^The term ‘research capital’ has also been used in relation to financial services and to describe the physical infrastructure for research, *e.g.*, the Department of Employment and Learning has a ‘Research Capital Investment Fund’ which supports physical refurbishment and infrastructure of buildings dedicated to research.

^b^In addition is the concept of ‘intellectual capital’ which is “created through a combination and exchange of existing intellectual resources, which may exist in the form of explicit and tacit knowledge and knowing capability” Nahapiet & Ghoshal: Social Capital, Intellectual Capital, and the Organisational Advantage. In Lesser EL. *Knowledge and Social Capital: Foundations and Applications.* Oxford: Butterworth Heinemann; 2000, pp. 131.

^c^There are times when one group, *e.g.*, the practitioner group may also contain clinical academics but these tend to occur in university hospitals where the link between the two groups has been bridged.

## Abbreviations

EoE: East of England; NIHR: National Institute for Health Research; NIHR RDS: NIHR Research Design Service.

## Competing interests

The authors declare they have no competing interests.

## Authors’ contributions

This article draws on our experiences of working for the NIHR Research Design Service for the East of England. GG is the current Director and MR was the Deputy Director until March 2012. GG drew upon her sociological knowledge of ‘social capital’ to develop the concept of ‘research capital’ and MR drew upon her knowledge of organisations and systems to show how the concept is operationalized. Both authors read and approved the final manuscript.
